# A protocol for a systematic review of behaviour change techniques used in the context of stillbirth prevention

**DOI:** 10.12688/hrbopenres.13375.1

**Published:** 2021-08-19

**Authors:** Tamara Escañuela Sánchez, Molly Byrne, Sarah Meaney, Keelin O'Donoghue, Karen Matvienko-Sikar

**Affiliations:** 1 Pregnancy Loss Research Group, Deparment of Obstetrics and Gynaecology, University College Cork, Cork, Ireland; 2 INFANT Centre, University College Cork, Cork, Ireland; 3 Health Behaviour Change Research Group, School of Psychology, NUI Galway, Galway, Ireland; 4 National Perinatal Epidemiology Centre, University College Cork, Cork, Ireland; 5 School of Public Health, University College Cork, Cork, Ireland

**Keywords:** stillbirth, intervention, behaviour change, risk factors

## Abstract

**Background:** Stillbirth is a devastating pregnancy outcome that affects approximately 3.5 per 1000 births in high-income countries. Previous research has highlighted the importance of focusing prevention efforts on targeting risk factors and vulnerable groups. A wide range of risk factors has been associated with stillbirth before, including maternal behaviours such as back sleep position, smoking, alcohol intake, illicit drug use, and inadequate attendance at antenatal care. Given the modifiable nature of these risk factors, there has been an increase in the design of behaviour change interventions targeting such behaviours to reduce the risk of stillbirth.

**Objectives:** The aim of this study is to identify all behavioural interventions with a behavioural component designed and trialled for the prevention of stillbirth in high-income countries, and to identify the behaviour change techniques (BCTs) used in such interventions using the Behaviour Change Techniques Taxonomy V1 (BCTTv1).

**Inclusion criteria:** Interventions will be included in this review if they (1) have the objective of reducing stillbirth rates with a focus on behavioural risk factors; (2) are implemented in high-income countries; (3) target pregnant women or women of childbearing age; and (4) are published in research articles.

**Methods:** A systematic search of the literature will be conducted. The results of the search will be screened against our inclusion criteria by two authors. The following data items will be extracted from the selected papers: general information, study characteristics, participant and intervention/approach details. The Cochrane Effective Practice and Organization of Care (EPOC) risk of bias criteria will be used to assess the methodological quality of included studies. Intervention content will be coded for BCTs as present (+) or absent (-) by two authors using the BCTTv1, discrepancies will be discussed with a third author. A narrative synthesis approach will be used to present the results of this systematic review.

## Abbreviations

PRISMA: Preferred Reporting Items for Systematic Reviews and Meta-Analyses; BCT: Behaviour Change Technique.

## Introduction

Stillbirth is one of the most devastating outcomes of pregnancy that expectant parents can face. In Ireland, a stillbirth is defined as an infant born weighing 500 grams or more or at a gestational age of at least 24 weeks who shows no signs of life ^1^. However, this definition of stillbirth is not globally accepted, with different countries using different weight or gestational thresholds, ranging from 350 grams to 1000 grams and from 20 weeks to 28 weeks ^2^. The worldwide estimates of stillbirth rates in 49 high-income countries at 28 weeks gestation was 3.5 per 1,000 total births in 2015, with country-specific rates varying from 1.3 in Iceland to 8.8 in Ukraine ^3^. In Ireland, the latest data published by the National Perinatal Epidemiology Centre shows that the prevalence of stillbirth was 3.8 per 1,000 births in 2017 ^4^.

The stillbirth series published in the Lancet in 2016 highlighted the importance of focusing future stillbirth prevention efforts in high-income countries on targeting specific causes, risk factors, and vulnerable groups ^5^. In high-income countries, the majority of stillbirths occur prior to labour and are associated with placental pathology ^6^. However, previous research has shown that stillbirth is associated with a wide range of risk factors, including maternal medical factors, for example hypertensive disorders or diabetes ^7– 9^; factors associated with the woman’s obstetric history, for example having a history of previous pregnancy loss ^10^, primiparity ^11^ or multiple pregnancy ^12^; pregnancy related complications such as placental insufficiency ^13^ or fetal growth restriction ^14^; ethnic ^15^ and socioeconomic status ^16^; maternal overweight and obesity ^17– 19^; and maternal age ^20– 22^. Maternal behavioural risk factors include factors such as sleep position ^23^, smoking ^5, 24, 25^, alcohol intake ^26, 27^, illicit drug use ^28– 31^ and inadequate antenatal care ^32, 33^. Such behavioural risk factors are modifiable and so provide useful targets for stillbirth prevention interventions.

Considering that some of the mentioned maternal behavioural risk factors have the potential to be modified, there has been an increase in development and implementation of antenatal behavioural interventions targeting those behaviours to reduce the risk of stillbirth. Behavioural interventions are defined here as those targeting behaviours such as smoking cessation interventions, midwifery led care, birth attendant training, alternative packages of antenatal care and diet and exercise interventions ^34^. However, in an overview of 43 Cochrane reviews assessing 61 different stillbirth prevention approaches across the globe, few reviews were found to produce clear evidence as the effectiveness of interventions differed across settings, highlighting the importance to understand the intervention’s context ^34^.

Previous studies have been conducted to explore facilitators and barriers influencing health behaviours during pregnancy ^35– 37^. Identifying and synthesising all existing behaviour change interventions designed in the context of stillbirth prevention, combined with the information obtained from the literature exploring factors influencing behavioural risk factors ^35– 37^, will inform the development of new strategies applicable in high-income countries. Given the variability across definitions, outcomes and measures of stillbirth, and the potential for differential intervention approaches to be used, there is a need to identify and synthesise the different components, or behaviour change techniques (BCTs), used in such interventions. A BCT is an observable, replicable and irreducible component of an intervention, an “active ingredient”, designed to alter or redirect casual processes that regulate behaviour ^38^. Identification of BCTs in interventions allows for accurate replication of interventions and faithful implementation of interventions demonstrating effectiveness ^38^. Hence, knowing which specific BCTs have been used within interventions is important to build cumulative evidence towards delivering effective and replicable interventions in this context. Additionally, reviewing and identifying BCTs in stillbirth prevention interventions will also enable future investigations of links between BCTs and mechanisms of action that will facilitate the optimisation of intervention effectiveness ^39^.

To our knowledge, there has been no systematic examination of BCTs used in behaviour change interventions designed with the objective to reduce stillbirth risks. Such a review is essential to informing and improving the development of future stillbirth prevention interventions. This research will build on previous reviews of general stillbirth prevention interventions (including infection management, pharmacotherapy for prevention of pre-eclampsia, screening for diabetes, ultrasound in early and late pregnancy, antenatal cardiotocography for fetal assessment), such as the one conducted by Ota and colleages ^34^, by focusing on those interventions with a behavioural component and by aiming to identify the BCTs used within those interventions.

The aim of this systematic review is to identify all research studies examining behaviour change interventions used in the context of stillbirth prevention within high-income countries, and to synthesise the BCTs used in these interventions.

### Objectives

• To identify behavioural interventions used for the prevention of stillbirth in high-income countries.
• To identify and code the BCTs used in such interventions using the Behaviour Change Techniques Taxonomy V1 (BCTTv1) ^38^.

## Protocol

Details of this review have been submitted for registration to the PROSPERO database (submitted 05/07/2021). Details of the PROSPERO protocol registration will be provided in the final systematic review article. Amendments made to the protocol will be acknowledged on PROSPERO and in any publications following this study. This protocol has been informed by the Preferred Reporting Items for Systematic Review and Meta-Analysis Protocols (PRISMA-P) guidelines for the reporting of systematic reviews ^40^.

### Eligibility criteria

The “PICO” framework was used to select inclusion and exclusion criteria for this review ( Table 1). For the purpose of this review, we did not select any specific definition for stillbirth because, as noted, definitions differ internationally ^2^.

**Table 1.** PICO framework.

PICO framework	Eligibility criteria
Population or problem	Population: - Pregnant women - Women of reproductive age Setting: - High-income countries
Intervention or exposure	Any intervention published in research articles designed to prevent stillbirth that includes a focus on behavioural risk factors.
Comparison	Participants who were not exposed to an intervention or who receive 'standard care'
Outcome	Behaviour change techniques used in the interventions, classified using the Behaviour Change Technique Taxonomy V1 ^38^

The following study types are eligible for inclusion: case-control studies, randomised control trials, cross-sectional studies, and quasi experimental studies. Studies will be eligible for inclusion when published in high-income countries given the differences between care systems and the challenges associate to pregnancy outcomes amongst low-, middle- and high-income countries. High-income countries will be defined based on the World Bank Country Classification (Gross National Income per capita of $12,696 or more in 2020) ^41^. The findings of this systematic review will help inform the development of an intervention to be used in a high-income country setting.

### Information sources

A systematic literature search covering all interventions in the context of stillbirth prevention from inception to present will be performed in the following databases: CINAHL Complete, SocIndex, Web of Science, PubMed, PsycINFO and Open Grey. Searches in Open Grey and contact with authors will facilitate access to non-published articles. Additionally, we will also conduct a manual reference list searching of the identified articles.

### Search strategy

Keyword searches will be used across four different concepts (1) Stillbirth, (2) Intervention, (3) Pregnancy and (4) Study design. The search strategy was developed for PubMed (see Table 2) but it will be adapted depending on the database in combination with database-specific filters.

**Table 2.** Example of search.

Database: Pubmed Date:		
	Search Terms	Search Results
Concept 1 Stillbirth		
**1.**	TI/AB Stillbirth	
**2.**	TI/AB Fetal death	
**3.**	TI/AB Intrauterine death	
**4.**	TI/AB Perinatal death	
**5.**	**S1 OR S2 OR S3 OR S4**	
Concept 2 Intervention		
**6.**	TI/AB Health promotion	
**7.**	TI/AB Education	
**8.**	TI/AB Awareness	
**9.**	TI/AB Information	
**10.**	TI/AB Behaviour	
**11.**	TI/AB Behavior	
**12.**	TI/AB Behaviour Change	
**13.**	TI/AB Behavior change	
**14.**	TI/AB Prevent*	
**15.**	TI/AB Intervention	
**16.**	TI/AB Strategy	
**17.**	TI/AB Guidance	
**18.**	**S6 OR S7 OR S8 OR S9 OR S10 OR S11 OR S12** **OR S13 OR S14 OR S15 OR S16 OR S17**	
Concept 3 Pregnancy		
**19.**	TI/AB Pregnant women	
**20.**	TI/AB Pregnancy	
**21.**	TI/AB Childbearing age	
**22.**	TI/AB Prenatal	
**23.**	TI/AB antenatal	
**24.**	**S19 OR S20 OR S21 OR S22 OR S23**	
Concept 4 Type of report		
**25.**	TI/AB “Randomised controlled trial”	
**26.**	TI/AB “Randomized controlled trial”	
**27.**	TI/AB RCT	
**28.**	TI/AB “control group”	
**29.**	TI/AB “controlled trial”	
**30.**	TI/AB “case-control study”	
**31.**	TI/AB “quasi-experimental”	
**32.**	TI/AB Initiative	
**33.**	TI/AB Guideline	
**34.**	**S25 OR S26 OR S27 OR S28 OR S29 OR S30** **OR S31 OR S32 OR S33**	
**35.**	**S5 AND S18 AND S24 AND S34**	
Limiters: - Humans		

### Study records

**Data management**

Studies will be imported into Endnote, duplicates will be identified by using the automatic “Check for Duplicates” tool, as well as manually screening the results. The screening of titles will be conducted using Rayyan, which is a web platform for systematic reviews ^42^.

**Selection process**

Two independent reviewers (TES, KMS) will individually screen titles, abstracts, and full articles to identify eligible studies, using our inclusion and exclusion criteria. In cases of uncertainty in title and abstract screening, studies will be included in the full text review stage. Discrepancies will be resolved through consensus discussion and/or recourse to a third reviewer.

A PRISMA flow diagram ^43^ will be used to show process of study screening and summarise the inclusion and exclusion criteria at each stage of the review.

**Data collection process**

Once the final list of studies to include in the review has been determined, all supplementary materials or additional studies related to the same intervention will be retrieved by TES before data extraction or quality assessments. When direct access is not provided to these supplementary materials online, the original study authors will be contacted to request these materials. Published manuscripts of trial outcomes and intervention protocols will be examined for intervention details.

### Data items

A structured data extraction sheet using Microsoft Word will be used to extract the study characteristics (see *Extended data* ^44^), which will be piloted in advanced. Data will be extracted by one reviewer (TES) and verified for accuracy by another reviewer (KMS).

Study characteristics to be extracted include:

(1) General information: Authors, title, name of intervention/approach (if applicable), year of publication, country of origin.
(2) Study characteristics: Study aims/objectives, type of study/report, stillbirth definition used, recruitment strategy.
(3) Participants: cohort size, number of people in control arm (if applicable), number of people in intervention arm (if applicable), mean age, age range, socio-economic status, education level, ethnicity, pregnancy status, relationship status.
(4) Intervention/approach details: setting, investigated topic/target behaviour, description of intervention/approach and control treatments, delivery mode, intervention duration, current stage of implementation, outcomes measured in intervention (e.g. reduction stillbirth rates, increased awareness, behaviour change), effectiveness of intervention.

### Outcomes and prioritization

Primary outcome:

- BCTs used (as described in the BCTTv1 ^38^)

### BCT coding

The coding of the BCTs included in the identified interventions/approached will be conducted following the BCTTv1 approach ^38^. Intervention content will be coded for presence (+) or absence (-) of the BCTs. This process will be done by two authors independently (TES, KMS) and discussion and comparison will be used to address any discrepancies. A third member of the research team (MB) will be consulted in case of disagreement. The findings of the BCT coding will be presented in tabular format.

### Risk of bias in individual studies/quality assessments

The Cochrane Effective Practice and Organization of Care (EPOC) risk of bias criteria ^45^ will be used to assess the methodological quality of the included studies that will subsequently feed into the GRADE process ^46^.

### Data synthesis

We anticipate that a meta-analytic approach would be inappropriate for comparison of studies due to the potential high heterogeneity of the interventions and potential BCTs. Hence, we will use a narrative synthesis approach as recommended by Cochrane when a meta-analytical approach is not possible, to summarise the findings of BCTs. The findings will also be presented in tabular form. This approach will facilitate the description of the interventions and BCTs used, taking into account quality appraisals.

### Analysis of subgroups or subsets

While subgroup analyses may be undertaken, it is not possible to specify the groups in advance.

### Dissemination

The PRISMA checklist ^43^ will be used to report findings of the review. We will communicate the findings by publication in a peer-reviewed journal, and by participation in scientific meetings and national and international conferences.

## Study status

Study protocol has been completed.

Database search completed.

Title screening completed.

Conducting abstract screening.

## Data availability

### Underlying data

No underlying data are associated with this article.

### Extended data

Harvard Dataverse: Study Characteristics extraction table for a protocol for a systematic review of behaviour change techniques used in the context of stillbirth prevention. https://doi.org/10.7910/DVN/0GCWXX ^44^.

### Reporting guidelines

Harvard Dataverse: PRISMA-P Checklist for “A protocol for a systematic review of behaviour change techniques used in the context of stillbirth prevention". https://doi.org/10.7910/DVN/6WNOFO ^47^.

Data are available under the terms of the Creative Commons Zero "No rights reserved" data waiver (CC0 1.0 Public domain dedication).
