# Supplementary material for: A protocol for a systematic review of behaviour change techniques used in the context of stillbirth prevention
Source: HRB Open Res. 2022 Mar 11;4:92. Originally published 2021 Aug 19. [Version 2] doi: 10.12688/hrbopenres.13375.2 (PMC9874168; doi:10.12688/hrbopenres.13375.2)
Supplement: Peer review history

# Peer review: Reviewer response for version 2

**DOI:** 10.21956/hrbopenres.14760.r33274

**Published:** 2023-01-24

**Authors:** Andrea M. Patey

**Affiliations:** 1 Centre of Implementation Research, Ottawa Hospital Research Institute, Ottawa, ON, Canada; 2 School of Epidemiology and Public Health, University of Ottawa, Ottawa, ON, Canada

The Authors provide a detailed protocol to identify all behavioural interventions with a behavioural component designed and trialled for the prevention of stillbirth in high-income countries, and to identify the behaviour change techniques (BCTs) used in such interventions using the Behaviour Change Techniques Taxonomy V1 (BCTTv1). They efficiently provide rationale for this review in that there has not been a systematic review of behaviour change interventions conducted in the context of stillbirth prevention. The Authors argue that the review is important to understand what techniques have been used previous to inform the development of theoretically robust evidence based interventions.

 I believe that the authors have appropriately addressed concerns raised by previous reviewers and have provided strong methods for their investigation to address their proposed objectives. I have no major or minor concerns with the protocol.

Is the study design appropriate for the research question?

Yes

Is the rationale for, and objectives of, the study clearly described?

Yes

Are sufficient details of the methods provided to allow replication by others?

Yes

Are the datasets clearly presented in a useable and accessible format?

Yes

Reviewer Expertise:

Implementation science, behaviour science and health psychology

I confirm that I have read this submission and believe that I have an appropriate level of expertise to confirm that it is of an acceptable scientific standard.

# Peer review: Reviewer response for version 2

**DOI:** 10.21956/hrbopenres.14760.r31640

**Published:** 2022-04-22

**Authors:** Rae Thomas

**Affiliations:** 1 Institute for Evidence-Based Healthcare, Bond University, Gold Coast, Australia

Thank you to the authors for providing clarity to my queries. I am happy with the authors' responses.

Is the study design appropriate for the research question?

Partly

Is the rationale for, and objectives of, the study clearly described?

No

Are sufficient details of the methods provided to allow replication by others?

No

Are the datasets clearly presented in a useable and accessible format?

Not applicable

Reviewer Expertise:

I have conducted multiple systematic reviews and have used BCTs and implementation strategies to design interventions.

I confirm that I have read this submission and believe that I have an appropriate level of expertise to confirm that it is of an acceptable scientific standard.

# Peer review: Reviewer response for version 1

**DOI:** 10.21956/hrbopenres.14568.r30847

**Published:** 2021-12-16

**Authors:** Rae Thomas

**Affiliations:** 1 Institute for Evidence-Based Healthcare, Bond University, Gold Coast, Australia

Thank you for the opportunity to review this systematic review protocol. The authors are hoping to identify behavioural intervention to prevent still birth and determine whether these interventions used behaviour change techniques. It is a very interesting topic and I wish the authors well. I have a few comments for them to consider and hope the review is not too far along for these comments to be of assistance.

**Introduction**:

 It was difficult to ascertain the necessity for this review from the introduction. The authors rightly mention there are already interventions that target some of the modifiable risk factors associated with still birth but that the results from a Cochrane review of 61 different still birth approaches conducted in 2020 found discrepancies across different settings. This was justified as a need to understand an intervention’s context. Does this mean different countries? The authors also mention published studies that specifically explore barriers and facilitators that influence health behaviours during pregnancy.

 I assume this systematic review is a likely precursor to an intervention trial. If this is the case, I am not sure the argument has been made that this new systematic review is different to other reviews or adds extra information. Could the authors not extract the studies from the Cochrane review that relate to ‘high income countries’, identify the BCTs in these studies and then use the barriers and facilitator studies to create a logic model for some intervention trials of identified modifiable behaviours?

**Methods**:

 Why would the authors be interested in searching for Guidelines (line 33 of search strategy) for an intervention question?

 What is the justification of including most study designs (lines 25-31)? All study designs in the search strategy have comparison groups but the cross-sectional design in the eligibility criteria does not. How is this reconciled? If the authors are inclusive of all designs, do not add them to the search strategy.

 What is “initiative” (line 32)?

 Conducting a forward citation search of included studies would be beneficial.

 What proportion of data extraction will be “verified for accuracy”? I notice the coding of BCTs (present/absent) is elsewhere, but wouldn’t this happen at the data extraction phase?

 The EPOC risk of bias is appropriate for studies with a control group but what about the cross-section study design mention in the eligibility section? How many authors will independently conduct the RoB assessment?

 I do not understand how you would conduct a meta-analysis of presence or absence of BCTs regardless of high heterogeneity. Do the authors mean that they would examine the presence or absence of BCTs within the context of intervention effectiveness? Would it be the number of BCTs used?

 I apologise that I am unclear about the purpose and outcome of this protocol.

Is the study design appropriate for the research question?

Partly

Is the rationale for, and objectives of, the study clearly described?

No

Are sufficient details of the methods provided to allow replication by others?

No

Are the datasets clearly presented in a useable and accessible format?

Not applicable

Reviewer Expertise:

I have conducted multiple systematic reviews and have used BCTs and implementation strategies to design interventions.

I confirm that I have read this submission and believe that I have an appropriate level of expertise to confirm that it is of an acceptable scientific standard, however I have significant reservations, as outlined above.

# Response

**Published:** 2022-03-07

**Authors:** Tamara Escanuela Sanchez

**Affiliations:** University College Cork, Ireland

Thank you for taking the time to review out protocol and suggest improvements

**Reviewer comment 2.1:**

 It was difficult to ascertain the necessity for this review from the introduction. The authors rightly mention there are already interventions that target some of the modifiable risk factors associated with still birth but that the results from a Cochrane review of 61 different still birth approaches conducted in 2020 found discrepancies across different settings. This was justified as a need to understand an intervention’s context. Does this mean different countries? The authors also mention published studies that specifically explore barriers and facilitators that influence health behaviours during pregnancy.

*Response to comment 2.1:*

 The focus of this review is to explore interventions that have a behavioural change component, targeting known modifiable risk factors for stillbirth and learning what the components of these interventions are.

 We aim to gather enough evidence and theoretical foundations to inform the development of a behaviour change intervention that has an impact on stillbirth prevention. Hence, the objective of this review is to understand behaviour change techniques have been used so far in the context of stillbirth prevention interventions with a behaviour change component.

 We do not aim to look at differences between countries, but to focus on high-income settings in order to gather evidence relevant to the context of the review.

**Reviewer comment 2.2:**

 I assume this systematic review is a likely precursor to an intervention trial. If this is the case, I am not sure the argument has been made that this new systematic review is different to other reviews or adds extra information. Could the authors not extract the studies from the Cochrane review that relate to ‘high income countries’, identify the BCTs in these studies and then use the barriers and facilitator studies to create a logic model for some intervention trials of identified modifiable behaviours?

*Response to comment 2.2:*

 The aim of this review is to explore behaviour change interventions targeting modifiable risk factors for stillbirth.

 The Cochrane review has a broader objective than the our review, as it looked at a range of interventions, not just those with a behavioural focus. To date, there has not been any systematic review of behaviour change interventions conducted in the context of stillbirth prevention. Such a review is needed to understand what techniques have been used previously to inform the development of theoretically robust evidence based interventions.

 We are aware of and acknowledge the usefulness of the Cochrane review and anticipate that interventions included in the Cochrane review will also be identified in our search. However, the search proposed in our review will also enable us to identify additional studies published since the Cochrane review search, in addition to any additional, specific behaviour change interventions relevant to stillbirth. We will manually check all of the interventions included in systematic reviews identified in our search –included the named Cochrane review- to ensure that all potentially relevant interventions are included.

 We consider then that relying on the Cochrane review only would not meet our aims due to the aim of the review to provide a comprehensive up-to-date review of interventions including behavioural components to prevent stillbirth.

 We have added further explanation to our introduction section to clarify this point further (Page 3, paragraph 2):

*Further, this Cochrane review of reviews had a focus on a broad range of interventions (i.e., nutritional interventions, prevention and management of infection, prevention and management of morbidities, screening and management of fetal growth), not only those with a behaviour change component. To date, there has not been any systematic review of behaviour change interventions conducted in the context of stillbirth prevention. Hence, this review is important to understand what techniques have been used previous to inform the development of theoretically robust evidence based interventions.*

**Reviewer comment 2.3:**

 Why would the authors be interested in searching for Guidelines (line 33 of search strategy) for an intervention question?

*Response to comment 2.3:*

 Guidelines are included in our search strategy because we hypothesised that guidelines may include stillbirth prevention strategies involving behaviour components that are targeted at population level. 

**Reviewer comment 2.4:**

 What is the justification of including most study designs (lines 25-31)? All study designs in the search strategy have comparison groups but the cross-sectional design in the eligibility criteria does not. How is this reconciled? If the authors are inclusive of all designs, do not add them to the search strategy.

*Response to comment 2.4:*

 As we are being inclusive of public health guidelines and other population level approaches, such as public health campaigns, it is appropriate to include cross-sectional study designs. This is because, such approaches to stillbirth prevention at population level cannot easily be examined using experimental approaches and so may necessitate approaches such as cross-sectional studies

**Reviewer comment 2.5:**

 What is “initiative” (line 32)?

*Response to comment 2.5:*

 The term ‘initiative’ is operationalised in the current review as a plan or program that is intended to solve a problem. For example, the RCOG’s Each Baby Counts initiative which is a national quality improvement program to reduce the number of babies who die. https://www.rcog.org.uk/eachbabycounts

 We have added this term to our search to be inclusive of population level approaches, as noted previously.

**Reviewer comment 2.6:**

 Conducting a forward citation search of included studies would be beneficial.

*Response to comment 2.6:*

 Thank you for your suggestion. We will conduct a forward citation search of included studies. This has now been added to the protocol manuscript (page 8, 1rst paragraph):

*When identifying systematic reviews, a manual search of their included interventions will be conducted to ensure that all relevant interventions are included in this review. We will also perform a further manual citation search of included studies. Discrepancies will be resolved through consensus discussion and/or recourse to a third reviewer.*

**Reviewer comment 2.7:**

 What proportion of data extraction will be “verified for accuracy”? I notice the coding of BCTs (present/absent) is elsewhere, but wouldn’t this happen at the data extraction phase?

*Response to comment 2.7:*

 50% of the data extraction process will be verified by a senior researcher (KMS). If any issues of discrepancies are observed, the rest of the data extraction will also be checked for accuracy.

 The last phase of our screening process, which involves a very detailed exploration of the study using our inclusion and exclusion criteria, will also lead to discard some of the interventions.

 The BCT coding will be conducted afterwards on the interventions that meet our inclusion criteria. BCT coding forms the data synthesis phase of the review, whereby we will use the data extracted on the interventions used to code for behaviour change techniques. Coding is not done therefore during the data extraction phase because it is the next systematic stage of the review.

**Reviewer comment 2.8:**

 The EPOC risk of bias is appropriate for studies with a control group but what about the cross-section study design mention in the eligibility section? How many authors will independently conduct the RoB assessment?

*Response to comment 2.8:*

 The EPOC risk of bias tool assessing both randomised trials (and/or non-randomised trials and/or controlled before-after (CBA) studies) and interrupted time series (ITS) studies.

 Thank you for bringing that point up to our attention. After consideration, we have decided that the JBI critical appraisal checklist for both descriptive and analytical cross-sectional studies.

 Two authors will independently conduct the quality assessment.

**Reviewer comment 2.9:**

 I do not understand how you would conduct a meta-analysis of presence or absence of BCTs regardless of high heterogeneity. Do the authors mean that they would examine the presence or absence of BCTs within the context of intervention effectiveness? Would it be the number of BCTs used?

*Response to comment 2.9:*

 A meta-analysis study can not be conducted due to the heterogeneity of the interventions in terms of target behaviour, outcomes and BCTs used. We anticipate that trying to merge multiple behaviours and constructs, from a possibly small number of trials to fit into categories for a meta-analysis, would not provide reliable results into effectiveness. We apologise for the lack of clarity, we have revised the manuscript to further clarify this.

 We will examine the presence or absence of BCTs by coding the different elements of the interventions into the BCTTv1 taxonomy. Hence, we will not only provide information on the total number of BCTs used in each intervention but also on which specific BCTs have been used.

 The effects of the interventions for each behaviour will be summarised and presented briefly. The frequency of BCTs used in the context of effective or non-effective interventions will be presented in a tabular format and examined narratively.

**Reviewer comment 2.10:**

 I apologise that I am unclear about the purpose and outcome of this protocol.

*Response to comment 2.10:*

 We hope these clarifications and addition to the manuscript have clarified the purpose and outcomes of this study. Thank you for your comments.

# Peer review: Reviewer response for version 1

**DOI:** 10.21956/hrbopenres.14568.r30070

**Published:** 2021-08-31

**Authors:** Samah Alageel

**Affiliations:** 1 Community Health Sciences Department, College of Applied Medical Sciences, King Saud University, Riyadh, Saudi Arabia

The authors have provided a well-written protocol to answer an important question, the results of this review will have great implications for the prevention of stillbirth. 

 I have few comments that the authors might consider:

1. The inclusion of high-income countries only is not well justified, the authors mention the differences in healthcare systems, however, due to the focus on health behaviours in this review, a clearer justification is needed. Or a reconsideration of including low and middle-income countries. 
2. The main aim of this review, based on the rationale, is to understand which BCTs would be useful to replicate in future interventions or to utilize in routine settings. Yet, this is not clear from the objectives. Only reporting which BCTs were used answers half of the question. We need to understand which is more likely to work. Also, if the aim is to understand effectiveness, then including intervention studies would only be suitable.
3. The focus on different behaviours that might provide benefits in preventing stillbirth requires a subgroup analysis of these behaviours and which BCTs work for each.
4. The others mentioned applying for PROSPERO, I suggest updating this protocol based on their reviews and add the registration number as well.
5. A meta-analysis and a meta-regression would be useful to examine the effectiveness of these interventions and BCTs in reducing stillbirths.  

Is the study design appropriate for the research question?

Yes

Is the rationale for, and objectives of, the study clearly described?

Yes

Are sufficient details of the methods provided to allow replication by others?

Yes

Are the datasets clearly presented in a useable and accessible format?

Yes

Reviewer Expertise:

Behaviour change and disease prevention

I confirm that I have read this submission and believe that I have an appropriate level of expertise to confirm that it is of an acceptable scientific standard, however I have significant reservations, as outlined above.

# Response

**Published:** 2022-03-07

**Authors:** Tamara Escanuela Sanchez

**Affiliations:** University College Cork, Ireland

Thank you for taking the time to review and provide feedback on our protocol paper.

**Reviewer comment 1.1:**

 The inclusion of high-income countries only is not well justified, the authors mention the differences in healthcare systems, however, due to the focus on health behaviours in this review, a clearer justification is needed. Or a reconsideration of including low and middle-income countries. 

*Response to comment 1.1:*

 Thank you for your comment.

 We appreciate the relevance of identifying interventions used in middle- and low-income countries. However, even though we are exploring health behaviours in this review, the main focus is associated with stillbirth prevention.

 The challenges associated with decreasing the rates of stillbirth in middle- and low-income countries are very different to those in high-income countries as the local health systems context is crucial for preventing stillbirth. 98% of the stillbirths occurred every year are in low- and middle-income countries, and 55% of those are in rural families where skilled attendance and caesarean sections are much lower than for urban births (Lawn *et al.*, 2011). We also know from the data exposed in the stillbirth series published in the Lancet in 2011, that in high-income countries, intrapartum stillbirth rates are typically less than 0.5 per 1000 total births, compared with rates of 12 per 1000 total births in many middle- and low-income countries. According to the authors, most of the babies who die during labour are term babies whose deaths are often associated with suboptimal care. To prevent antepartum stillbirth, improved maternal health ad care during pregnancy is needed, whereas to prevent intrapartum stillbirths, better obstetric care is necessary (Lawn *et al*., 2011).

 Hence, given these differences, we consider that different reviews for the different contexts should be conducted to provide contextually relevant information that might be used to inform the development of future interventions. In our case, and given the scope and aims of our project, we made the decision of investigating high-income counties.

**Reviewer comment 1.2:**

 1.2 The main aim of this review, based on the rationale, is to understand which BCTs would be useful to replicate in future interventions or to utilize in routine settings. Yet, this is not clear from the objectives. Only reporting which BCTs were used answers half of the question. We need to understand which is more likely to work. Also, if the aim is to understand effectiveness, then including intervention studies would only be suitable. A meta-analysis and a meta-regression would be useful to examine the effectiveness of these interventions and BCTs in reducing stillbirths. The focus on different behaviours that might provide benefits in preventing stillbirth requires a subgroup analysis of these behaviours and which BCTs work for each.

*Response to comment 1.2:*

 We anticipate that the availability of data and the heterogeneity of outcomes, interventions and BCTs that the different reviewed papers explore would not allow strong statements about intervention effectiveness similar to a meta-analysis.

 Further, we consider that attempting to collapse multiple behaviours and constructs, from a potentially small number of trials, into categories for inclusion in a meta-analysis would not provide reliable results into intervention effects.

 As intervention effectiveness is not the main focus of this review, effects of interventions for each behaviour will be presented in table format and briefly narratively summarised. Intervention effects will be presented in terms of effect sizes where reported and/or as calculated from data presented within the reviewed papers.

**Reviewer comment 1.3:**

 The others mentioned applying for PROSPERO, I suggest updating this protocol based on their reviews and add the registration number as well.

*Response to comment 1.3:*

 Protocol has been updated according to PROSPERO reviews and registration ID has been added, thank you. Page 4 Section protocol:

*Details of this review have been submitted for registration to the PROSPERO database (ID CRD42021264914).*
